# “I was seen by a radiologist, but unfortunately I can’t remember the name and I still have questions. What should I do?” Radiologists should give thoughts to improve service professionalism and patient esteem

**DOI:** 10.1186/s40644-020-0292-7

**Published:** 2020-02-13

**Authors:** Andreas Gutzeit, Arne Fischmann, Rosemarie Forstner, Romana Goette, Bernhard Herzog, Claudia Kurtz, Chantal Hebler, Andrea Ladinger, Johannes M Froehlich, Janusch Blautzik, Orpheus Kolokythas, Simon Matoori, Sebastian Kos, Carolin Reischauer, Hubert Schefer, Peter Dubsky, Simon Peter Gampenrieder, Klaus Hergan, Wolfgang Gaissmaier, Dow-Mu Koh, Matthias Meissnitzer

**Affiliations:** 1 Department of Radiology, Paracelsus Medical University, Salzburg, Austria; 2 Department of Chemistry and Applied Biosciences, Institute of Pharmaceutical Sciences, ETH Zurich, Vladimir-Prelog-Weg 1-5 / 10, 8093 Zurich, Switzerland; 3 Institute of Radiology and Nuclear Medicine and Breast Center St. Anna, Hirslanden Klinik St. Anna, St. Anna-Strasse 32, 6006 Lucerne, Switzerland; 4 Institute of Radiology and Nuclear Medicine, Kantonsspital Luzern, Lucerne, Switzerland; 5 Department of Radiology, University of Washington, 1959 NE Pacific St, Seattle, WA 98190 USA; 6 Department of Medicine, University of Fribourg, Fribourg, Switzerland; 7 Department of Radiology, HFR Fribourg-Hôpital Cantonal, Fribourg, Switzerland; 8 Department of Oncology, Hirslanden Klinik St. Anna, St. Anna-Strasse 32, 6006 Lucerne, Switzerland; 9 Breast Center St. Anna, Hirslanden Klinik St. Anna, St. Anna-Strasse 32, 6006 Lucerne, Switzerland; 10 Department of Internal Medicine III with Haematology, Medical Oncology, Haemostaseology, Infectiology and Rheumatology, Oncologic Center, Salzburg Cancer Research Institute - Laboratory for Immunological and Molecular Cancer Research (SCRI-LIMCR), Paracelsus Medical University Salzburg, Muellner Hauptstraße 48, 5020 Salzburg, Austria; 11 Department of Psychology, University of Konstanz, P.O. Box 43, D-78457 Konstanz, Germany; 12 Cancer Research UK Clinical Magnetic Resonance Research Group, Institute of Cancer Research, Sutton, Surrey, UK

**Keywords:** Communication, Anxiety, Psychology, mammography

## Abstract

**Background:**

The aim of the study is to investigate how well patients remember the radiologist’s name after a radiological examination, and whether giving the patient a business card improves the patient’s perception of the radiologist’s professionalism and esteem.

**Methods:**

In this prospective and randomized two-centre study, a total of 141 patients with BI-RADS 1 and 2 scores were included. After screening examination comprising mammography and ultrasound by a radiologist, 71 patients received a business card (group 1), while 70 received no business card (group 2). Following the examination, patients were questioned about their experiences.

**Results:**

The patients in group 1 could remember the name of the radiologist in 85% of cases. The patients in group 2, in contrast, could only remember the name in 7% of cases (*p* < 0.001). 90% of the patients in group 1 believed it was very important that they are able to contact the radiologist at a later time, whereas only 76% of patients in group 2 felt that this was a very important service (*p* < 0.025). A total of 87% of the patients in group 1 indicated that they would contact the radiologist if they had any questions whereas 73% of the patients in group 2 would like to contact the radiologist but were not able to do so, because they could not remember the name (*p* < 0.001).

All questions were analysed with a Cochran-Mantel-Haenszel (CMH) test that took study centre as stratification into account. In some cases, two categories were collapsed to avoid zero cell counts.

**Conclusions:**

Using business cards significantly increased the recall of the radiologist’s name and could be an important tool in improving the relationships between patients and radiologists and enhancing service professionalism.

**Trial registration:**

We have a general approval from our ethics committee. The patients have given their consent to this study.

## Introduction

Radiological examinations are an indispensable part of modern medicine for the screening, diagnosis and follow-up of diseases. Owing to the increasing diagnostic speed and volume of imaging procedures, radiologists face significant reporting burdens and consequently less time to interact with patients [1–4]. Perhaps not surprising but detrimental to our profession, it was found that 76% of the patients did not know what the role of a radiologist is, with some believing that “radiologists are the guys who ask patients if somebody had allergies” [5].

There is an ongoing discussion within the radiological community as to whether the radiologist should be only an “imager” or whether he or she should be a more patient-oriented physician, undertaking active dialogues with patients concerning their health and disease management [6].

In a recently published study, 80–90% of radiologists did not directly talk to or engage with patients before or after the imaging studies because of the lack of time or uncertainty about its value [7, 8]. There is an ongoing discussion as to whether radiologists should rethink their role in the clinical care paradigms. “Radiologist 3.0” has emerged in the last few years as encouraging a shift from the “pure imager” to the patient-oriented specialist, whose job profile includes communicating radiology reports to the patients [9].

In our institutions, we have traditionally attached great importance to adequate communication with our patients, especially in the challenging field of mammography and breast ultrasound examination. However, we have noticed that despite our endeavours to discuss imaging results with our patients, a large number of them did not remember the radiologist’s name and had no idea how to reach the radiologist if they had further questions later on. One explanation is that the patients are so pre-occupied or stressed during these examinations that they cannot remember the names of the doctors or are afraid to ask.

The aim of this study is to investigate how well patients can remember the radiologist’s name under normal circumstances following breast mammographic and ultrasound examination, and the importance attached to being able to reach the radiologist afterwards if they have further questions or concerns. We also explored whether remembering the name of the radiologist, radiologist-patient bonding and patient satisfaction can be improved by giving each patient a business card after the radiological examination, with the aim of improving the service quality for patients.

## Material and methods

**Fig. 1:**
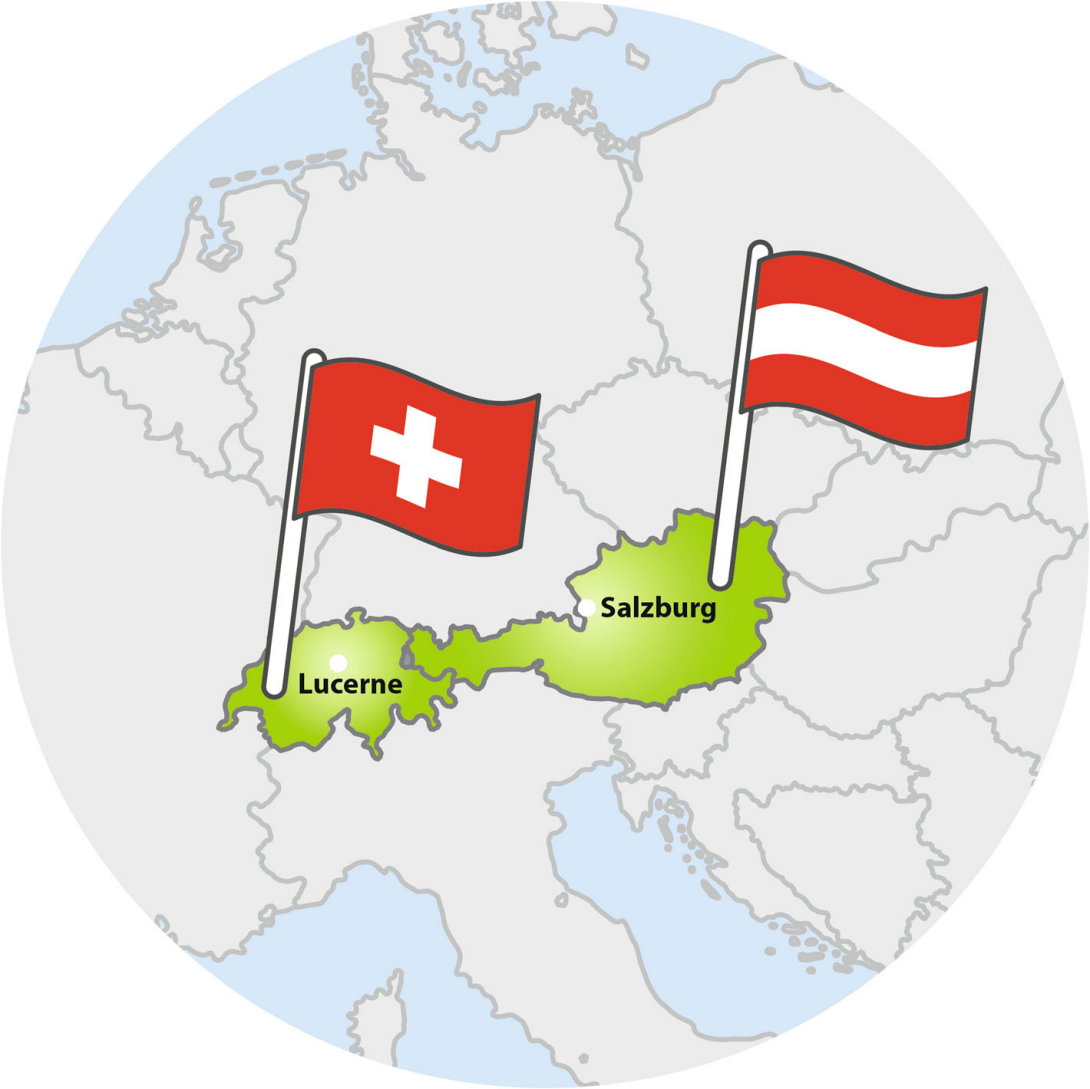
The radiologists from two breast imaging centers at the University Hospital Salzburg (Austria) and St. Anna Hospital in Lucerne (Switzerland) participated in this study

This prospective study was conducted from February 2019 to July 2019 in compliance with the Declaration of Helsinki. Signed written informed consent was obtained from the patients, who were evaluated anonymously for the participating physicians. This is a two-center study conducted at the University Hospital Salzburg (Austria) (Center 1) and St. Anna Hospital in Lucerne (Switzerland) (Center 2) (Fig. 1).

Centers 1 & 2 are certified breast imaging centers performing approx. 6000/ 7000 breast examinations per year respectively. The examinations in the study were exclusively carried out by Matthias Meissnitzer (MM) (Center 1) and by Andreas Gutzeit (AG) (Center 2). Both are board-certified radiologists with 9/19 years of experience in breast diagnostics, respectively. In addition, radiologist AG in Lucerne has been trained in communication psychology and has experience in designing psychological questionnaires as applied in this study.

All patients over 18 years of age who underwent routine imaging examination using mammography and ultrasound, for which the findings were categorized as BI-RADS 1 or 2 were prospectively recruited. All patients with conspicuous findings on additional ultrasound, those who could not be reached by telephone or refused to participate in the study were excluded. In the two centers, several specialized breast radiologists rotate. The patients did not know the radiologists from previous examinations.

The initial mammography was performed in Center 1/2 on a SenoClaire® Digital full field Breast 3D Tomosynthesis mammography/Siemens Healthcare mammography system. When the mammography (which was evaluated immediately by the radiologist) was completed, standard ultrasound examination was performed on a Voluson E8 machine, General Electric Healthcare/GE Logiq E10 system. These patients were randomly assigned to group 1 or 2 as described below.

### Group 1: patients given the radiologist’s business card after completion of the mammography and ultrasound examinations

After completing the mammogram examination, breast ultrasound was performed and the results were discussed with patients who fitted the inclusion/ exclusion criteria. At the beginning of the ultrasound examination, we greet each patient: “Good morning, my name is Dr. Meissnitzer (MM)/ Gutzeit (AG). I am your radiologist. Your mammography results are unremarkable. No suspicious abnormalities were detected. With your consent, I would like to perform ultrasound as well.”

**Fig. 2:**
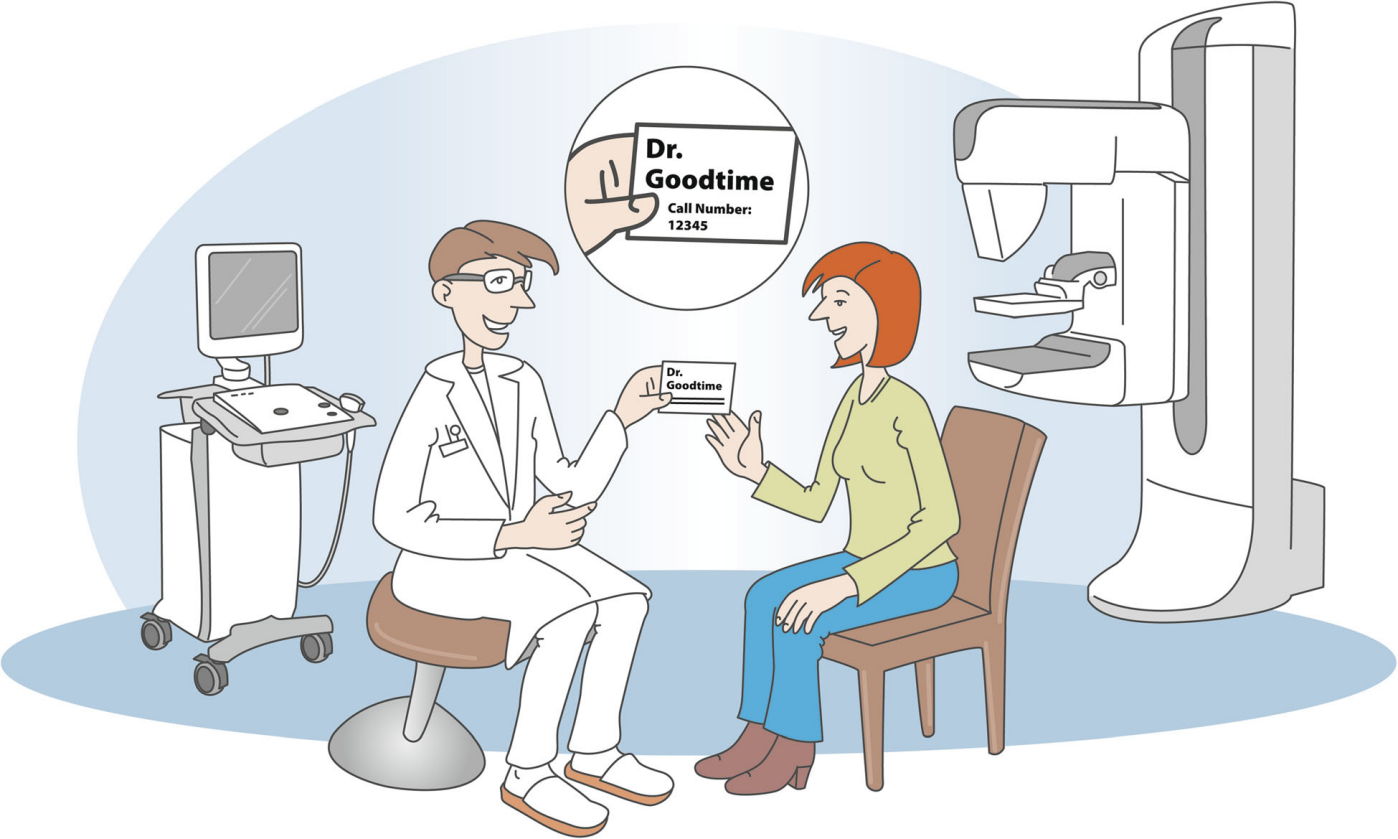
Following the mammography and ultrasound examinations, patients were given the radiologist’s business card (group 1)

At the end of the consultation, the radiologist bids goodbye to the patient with the words: “The ultrasound and mammography results were unremarkable. Do you have any further questions?” Following any discussions, the following words were added: “I would also like to give you my business card; and you are welcomed to contact me if you have any further questions. Goodbye!” (Fig. 2). For each consultation, the time expended for the ultrasound examination and patient dialogue was measured using a stopwatch. This was defined as the time period between opening the door to the examination room and when the patient entered, till when the patient/doctor left the room after the examination. The patients were unaware of the time measurements.

### Group 2: patients not given a business card after completion of the mammography and ultrasound examinations

After completion of the mammography, the ultrasound examination was performed with the same procedure as in group 1. At the beginning of the ultrasound examination, we greeted the patient: “Good morning, my name is Dr. Meissnitzer (MM)/Gutzeit (AG). I am your radiologist. Your mammography results were unremarkable. No suspicious abnormalities were detected. With your consent, I would now like to perform an ultrasound examination as well.”

**Fig. 3:**
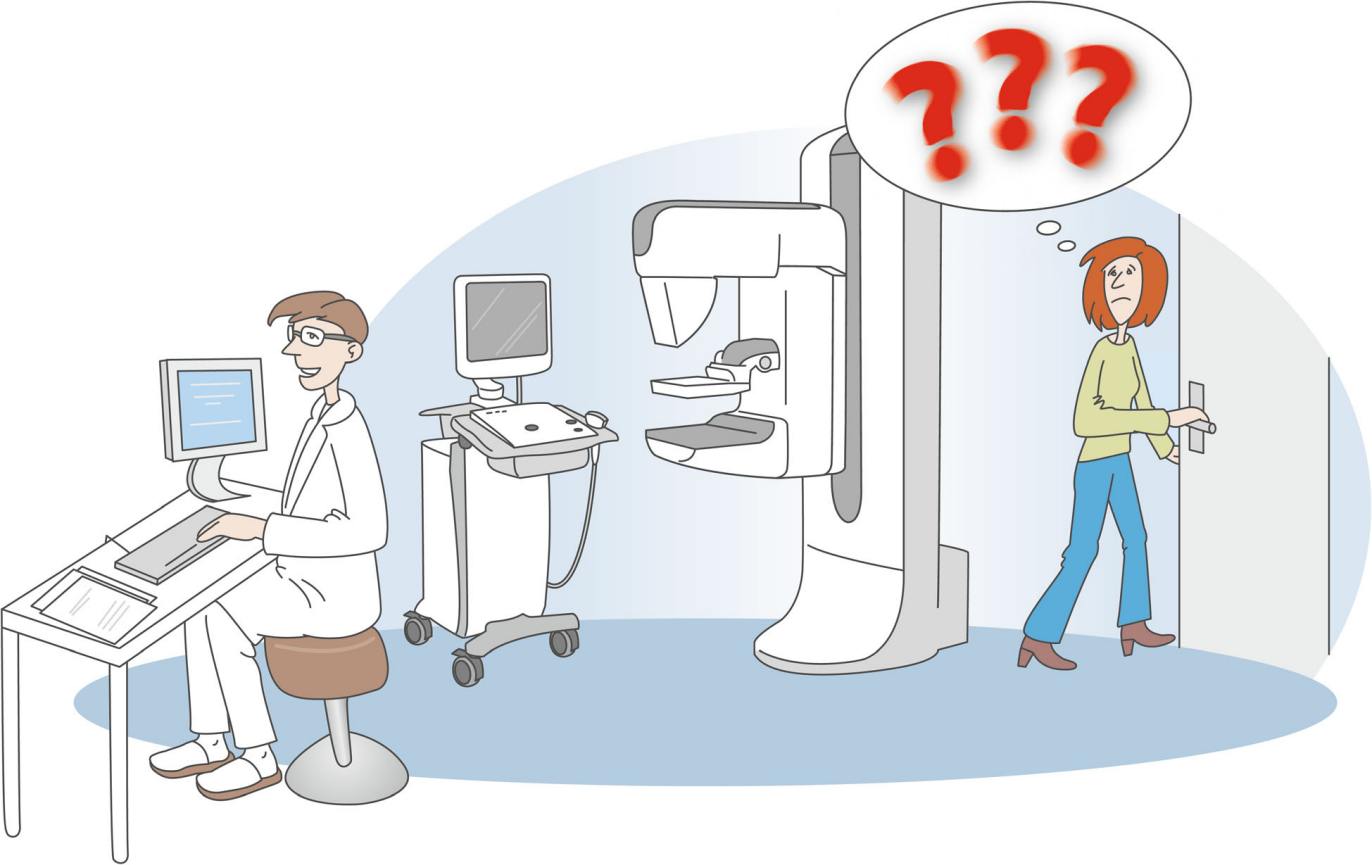
After completing the mammography and ultrasound examinations, the patient departs without receiving a business card from the radiologist (group 2)

At the end of the examination and discussion the results, we bid goodbye to the patient with the words: “Your ultrasound and mammography results were both unremarkable. This means they revealed no suspicious abnormalities. Do you have any further questions?”. The time expended for the patient dialogue and ultrasound examination was again measured using a stopwatch. No business card was given to the patients in this group (Fig. 3).

#### Telephone interview a few days after the examination

At 1–7 days after the imaging examinations, each patient received a telephone call from the research team. The interviews were conducted by two trained patient interviewers from our quality management staff (CH/AL). A total of 8 questions were asked and the responses recorded. In addition to the questions, the duration of the interview and the time of the interview relative to the imaging studies were documented. The questions, as well as the responses are summarized in Table 1.

**Table 1:** Summary of questions 1–8 and results. The numbers in questions 2,5,6 and 7 are divided into orders 1 to 5. The number 1 is the lowest and 5 the highest value

Questions	Answers	Group with business card (*N* = 71)	Group without business card (*N* = 70)	*p*-value
1. Do you remember the name of your radiologist?	Yes	60 (84.5%)	5 (7.1%)	< 0.001
	No	11 (15.5%)	65 (92.9%)	
2. How competent did the radiologist seem to you yesterday?	3	1 (1.4%)	4 (5.7%)	0.166
	4	4 (5.6%)	8 (11.4%)	
	5	66 (93.0%)	58 (82.9%)	
3. Would you recommend our radiology institution?	Yes	71 (100.0%)	70 (100.0%)	
	No	0 (0.0%)	0 (0.0%)	
4. If you could decide for yourself which radiology institution you would like to be examined in, would you come back to us?	Yes, I would only come to you in the future.	1 (1.4%)	4 (5.7%)	0.375
	I don’t care where my gynecologist sends me.	69 (97.2%)	65 (92.9%)	
	No, I’m going to go to another radiology institute.	1 (1.4%)	1 (1.4%)	
5. How important was the final discussion with the radiologist to you?	1	1 (1.4%)	0 (0.0%)*	0.668
	4	2 (2.8%)	5 (5.8%)	
	5	68 (95.8%)	65 (94.2%)	
6. How satisfied were you with our radiological services in general?	3	2 (2.8%)	0 (0.0%)*	0.310
	4	8 (11.3%)	6 (8.6%)	
	5	61 (85.9)	64 (91.4%)	
7. How important do you think is it to be able to contact the radiologist with questions in the future?	1	1 (1.4%)	1 (1.4%)	0.025
	2	1 (1.4%)	2 (2.9%)	
	3	0 (0.0%)*	9 (12.9%)	
	4	5 (7.0%)	5 (7.1%)	
	5	64 (90.1%)	53 (75.7%)	
8. Would you contact the radiologist again if you had any questions?	Yes	62 (87.3%)	5 (7.1%)	< 0.001
	No	5 (7.0%)	14 (20.0%)	
	I’d love to contact the radiologist, but I can’t remember his/her name.	4 (5.6%)	51 (72.9%)	

*For the Cochran-Mantel-Haenszel test, two categories were collapsed to avoid zero cell counts

#### Patient population

We approached 219 patients in both centers to take part in the study.

### Center 1 Salzburg

After the exclusion of 22 patients in Center 1 (19 patients could not be reached over the telephone, 3 rejected the study), 21 patients were included in group 1 (with business card) and 20 in group 2 (without business card) of our study.

### Center 2 Lucerne

We excluded 46 patients from Center 2 (32 patients could not be reached over the telephone, 4 rejected the study, 7 unexpectedly displayed a tumor on ultrasound examination, 3 patients showed poor language skills on the phone.) resulting in 100 patients being included from Center 2. A total of 50 patients were assigned to group 1 (with business card) and 50 patients to group 2 (without business card).

### Patients' inclusions from both centers

A total of 141 patients were included from the two centers: 71 with and 70 without business card. The mean patient age was 59 (range: 39 and 91 years).

### Questionnaire

The questions in the questionnaire (Table 1) were developed by an experienced radiologist with a degree in communication psychology (AG) and a quality manager with 10 years of experience in the development of patient questionnaires (CH).

#### Statistical analysis

All questions were analysed by a professional statistican (graf@biostatistics.ch) with a Cochran-Mantel-Haenszel (CMH) test that took study centre as stratification into account. In some cases, two categories were collapsed to avoid zero cell counts.

All analyses were performed in the R programming language (version 3.3.3) (R Core Team, 2017).

## Results

The ultrasound examinations and the discussion of the findings lasted on average 7.5 min (range 5.1–10.4). The duration of discussion did not differ between group 1 and 2 (*p* > 0.05).

The telephone interviews were conducted on average 2 days after the radiographic examination and lasted on average 3.6 min (range 1.7–22.0).

All questions and results are summarized in Table 1:

About 85% of the patients in group 1 could remember the radiologist’s name, compared with only 7% of the patients in group 2, (Question 1). In group 1, about 87% of patients said that they will contact the radiologist if they have any questions. In group 2, about 73% of patients said that they would like to contact the radiologist but would not remember his or her name (Question 8). There was no difference between the groups with regards to the perceived competence of the radiologist (question 2) (*p* = 0.166). There was also no difference as to whether the radiological institution is likely to be recommended by the patient (question 3 and 6). A total of 100% of the patients in both groups would recommend the radiology department to others.

In both groups, almost all patients indicated how important it was to be able talk to the radiologist (*p* = 0.668). Question 7 shows that in group 1 about 90% of the patients find it very important to be able to contact the radiologist in the future, whereas in group 2 only 76% of the patients stated this (*p* = 0.025).

### How many patients in group 1 (group with business card) have contacted the radiologist?

It was not the aim of the study to investigate how many patients actually called the radiologist after receiving business cards, but we confirm that not a single patient in this group actually wrote or emailed the radiologist.

## Discussion

In our study, radiological patients who are given a business card by the radiologist (group 1) following radiological examinations can remember the name of the radiologist in 85% of cases. Patients who were not given a business card after the examination (group 2) could only remember the radiologist’s name in 7% of cases (question 1), even though the radiologists introduce themselves to all patients before commencing the ultrasound studies. This difference was statistically significant (*p* < 0.001).

In group 1, 90% of the patients say they feel it is important that they can contact the radiologist at a later time (question 7). In group 2, on the other hand, only 76% feel that this is an important service (*p* < 0.025). In group 1 almost all patients (87%) say that they would contact the radiologist as a competent partner for questions (question 8) and in group 2 about 73% say they would like to contact the radiologist but are not able to because they didn’t remember the name. This difference was also statistically significant (*p* < 0.001). The remaining results show that the patients are able to manage their encounter with the radiologist even without knowing his or her name but feel equally well treated in radiological departments and independently perceive their attending radiologist as highly competent.

What does it mean that patients hardly remember the radiologist’s name after a normal examination? In the current health care system, there is an ongoing debate about patient-oriented medicine. This term is used to describe personalized medicine with strong human interactions and relationships. It is also called “humanized” medicine. In addition to providing psychological support for patients, patient-oriented medicine can improve the quality of care and lead to more efficient use of economic resources [10].

The main goal of radiology service is to perform and interpret high quality imaging examinations, as well as communicate the radiological results effectively to the referring physician or to the patient. The report must be accurate and easily understood. Reports should employ clear, unambiguous language [11]. In recent publications there has been an ongoing discussion about the role of radiologist in the clinical context. The question is: what should the position of the radiologist be? Should we be limited in our role as “pure imagers” or should the radiologist act as a more patient-oriented physician within the health care management system [12–24]? The future role of the radiologist is not discussed in detail in this publication. However, it seems clear that empathic care of the patient is only possible if the latter can call us when they have questions or concerns. However, if patients only remember the radiologist’s name in 7% of cases under normal conditions (group 2), this becomes impossible.

In question 7, 90% of patients say that the ability to ask the radiologist questions later on is an important aspect of their care, while in group 2 only 76% of patients consider this an important service.

Obviously, the patients have a need which they only become aware of when they are given a business card or the opportunity to ask for it. A recently published study described similar phenomena. In this study, it was shown that 81% of the patients who perceived the opportunity to discuss their imaging results with the radiologist rated this as a high value. On the other hand, when patients do not have the chance to discuss their results with a radiologist, only 14% of them perceive the contact with the radiologist as an important part of the overall service [25].

We asked ourselves why the names of the radiologists were so poorly remembered in group 2. We hypothesize that the patients may be extremely anxious during the breast examination, leading to poor memory. Furthermore, in the situation of an imaging examination, it may not seem important to the patient to ask for the radiologist’s name. We have had similar experiences with emergency patients in our hospital. When we ask who examined them in the emergency unit, most patients are unable to recall the name of the emergency doctors. We have not found any other studies on this subject, but suspect that our observations could be extrapolated to other medical specialties as well.

This study only examines the breast diagnostic section. The legitimate question is, what are the results in patients undergoing CT or MR examinations? According to the study by Gutzeit et al., direct communication after MRI examinations is clearly desired by patients [25]. Whether it economically  makes sense in the future to say goodbye to each patient after every single CT or MRI within the daily clinical routine has to be investigated. The mass of examinations in many institutes under today’s conditions is a realistic counterargument. On the other hand, the procedure could easily be transferred to general ultrasound and, according to our own study results, has been successfully implemented in our own institutes.

Another important point in the discussion of our results is the broad consensus in the radiological community that emerging artificial intelligence will change our discipline greatly in the coming decades [26]. We believe that radiologists need to show patients more what the value of a good radiology service is and that patients should perceive radiologists as unique experts. If we assume that machines will soon be better than radiologists, there is only one route left to us: we should not behave like machines, but become empathic doctors who are responsive to our patients’ needs before, during and after their examinations -- something which patients would highly value.

This study has numerous limitations: First, the examinations conducted in Centers 1 and 2 always involved the same two radiologists (AG and MM), one of whom has been trained in communication psychology (AG). Whether the results are broadly transferable to every radiologist or department would have to be investigated further. Second, the examinations were carried out only in the field of senology. In our experience, these patients may feel a greater burden of stress. The question of whether the recall of radiologists’ names may be equally poor in the group of patients who were not given business cards in other examination types would have to be examined further. Third, some radiologists may be concerned that they will be constantly called up or asked questions by patients who have been given a card. Although we have not been able to investigate this point further, we have noted that not a single patient, out of all the patients who were given business cards, has contacted us. However, this aspect would also have to be evaluated in further investigations. Fourth, at the beginning of the ultrasound examination, the doctor performing the exam knew whether he was going to end up giving the patient a card or not. As such, this was not a double-blind study. However, it is unlikely that a significant selection bias has occurred, since the examination time was not different between group 1 and 2. Scientifically, a double-blind study would have been better, but was practically challenging to organize.

## Conclusion

Under normal conditions, patients find it difficult to recall the name of the radiologists who undertake their imaging studies, and therefore may not be able to contact the radiologist for questions although they may like to do so. The act of giving the patients a business card significantly increases their recall of the radiologist’s name and could be an important factor in improving the relationships between patients and radiologists. The desire to be able to contact the radiologist when questions may arise later needs to be regarded as an important aspect of their care.

## Data Availability

Yes, all data are available.

## Abbreviation

BI-RADS: Breast Imaging Reporting and Data System
